# An integrative assessment to determine the genotoxic hazard of estuarine sediments: combining cell and whole-organism responses

**DOI:** 10.3389/fgene.2014.00437

**Published:** 2014-12-10

**Authors:** Pedro M. Costa, Miguel Pinto, Ana M. Vicente, Cátia Gonçalves, Ana P. Rodrigo, Henriqueta Louro, Maria H. Costa, Sandra Caeiro, Maria J. Silva

**Affiliations:** ^1^Departamento de Genética Humana, Instituto Nacional de Saúde Dr.Ricardo JorgeLisboa, Portugal; ^2^MARE – Marine and Environmental Sciences Centre/IMAR – Instituto do Mar, Departamento de Ciências e Engenharia do Ambiente, Faculdade de Ciências e Tecnologia da Universidade Nova de LisboaCaparica, Portugal; ^3^Departamento de Ciências e Tecnologia, Universidade Aberta, Rua da Escola PolitécnicaLisboa, Portugal; ^4^Centre for Environmental and Sustainability Research, Departamento de Ciências e Engenharia do Ambiente, Faculdade de Ciências e Tecnologia da Universidade Nova de LisboaCaparica, Portugal

**Keywords:** Comet assay, environmental risk assessment, sediment contamination, oxidative stress, HepG2 cells

## Abstract

The application of the Comet assay in environmental monitoring remains challenging in face of the complexity of environmental stressors, e.g., when dealing with estuarine sediments, that hampers the drawing of cause-effect relationships. Although the *in vitro* Comet assay may circumvent confounding factors, its application in environmental risk assessment (ERA) still needs validation. As such, the present work aims at integrating genotoxicity and oxidative DNA damage induced by sediment-bound toxicants in HepG2 cells with oxidative stress-related effects observed in three species collected from an impacted estuary. Distinct patterns were observed in cells exposed to crude mixtures of sediment contaminants from the urban/industrial area comparatively to the ones from the rural/riverine area of the estuary, with respect to oxidative DNA damage and oxidative DNA damage. The extracts obtained with the most polar solvent and the crude extracts caused the most significant oxidative DNA damage in HepG2 cells, as measured by the formamidopyrimidine-DNA glycosylase (FPG)-modified Comet assay. This observation suggests that metals and unknown toxicants more hydrophilic than polycyclic aromatic hydrocarbons may be important causative agents, especially in samples from the rural part of the estuary, where oxidative DNA damage was the most significant. Clams, sole, and cuttlefish responded differentially to environmental agents triggering oxidative stress, albeit yielding results accordant with the oxidative DNA damage observed in HepG2 cells. Overall, the integration of *in vivo* biomarker responses and Comet assay data in HepG2 cells yielded a comparable pattern, indicating that the *in vitro* FPG-modified Comet assay may be an effective and complementary line-of-evidence in ERA even in particularly challenging, natural, scenarios such as estuarine environments.

## INTRODUCTION

Ever since the original publication of the protocol by Singh et al. (1988), the alkaline Comet assay rapidly developed into one of the most prolific tools for those performing research on environmental genotoxicity. Indeed, this paramount technical achievement quickly became one of the most important tools to assess the hazards of genotoxicants in the environment, with emphasis on the aquatic milieu (see Mitchelmore and Chipman, 1998). Within these ecosystems, sediments have been targeted in environmental risk assessment (ERA) studies due to their ability to trap, store, and (depending on disruption of their steady-state) release contaminants back to the biota. The range of these substances includes genotoxicants, from metals to dioxins and polycyclic aromatic hydrocarbons (PAHs), the latter being highly hydrophobic mutagens and holding high affinity to organic matter and fine fraction (see Chen and White, 2004, for a review).

It is becoming increasingly common to employ *in vitro* approaches with fish cell lines exposed to aquatic sediment extracts to determine the genotoxic potential of bioavailable pollutants (for instance, Kosmehl et al., 2008; Yang et al., 2010; Šrut et al., 2011). In contrast, similar work with human cell lines is less common. The relatively simple logistics of *in vitro* assays renders their combination with the Comet assay appealing for the determination of the genotoxic effects of pollutants in sediment and water samples. In particular, the human hepatoma HepG2 cell line has long been regarded as metabolically competent to determine genotoxic effects of chemical substances, with proven sensitivity for the detection of such effects through the Comet assay (Uhl et al., 1999). Still, regardless of being logistics-friendly and able to reduce much of the confounding factors that often hinder the interpretation of results when testing or sampling *in situ* aquatic organisms, it is clear that the results obtained *in vitro* need to be compared with other lines-of-evidence in order to obtain practical validation for the purpose of ERA.

The analysis of biomarker responses related to oxidative stress is deemed to be indicative of reactive oxygen species (ROS) produced directly or indirectly as a consequence of exposure to xenobiotics. As such, oxidative stress biomarkers allow a pertinent approach to evaluate sub-individual effects of toxicological challenge and therefore enable an overall assessment of the effects of environmental contaminants or their mixtures (see, for instance, van der Oost et al., 2003; Picado et al., 2007). Oxidative-stress related biomarkers in vertebrate or invertebrates have been proposed for ERA under a multiplicity of scenarios, whether concerning specific substances, classes of substances or particularly challenging mixtures as aquatic sediments (e.g., van der Oost et al., 2003; Scholz et al., 2008; Bonnineau et al., 2012; Chapman et al., 2013). Nevertheless, biomarkers such as lipid peroxidation and the activity of anti-oxidant enzymes may be modulated by many confounding factors and by distinct types of both organic and inorganic toxicants, rendering difficult the determination of cause–effect relationships. This may be particularly critical when addressing complex contaminant matrices such as aquatic sediments (see Chapman et al., 2013, for a recent review). Still, as for other biomarker responses, measuring oxidative damage and defenses in wild organisms has long become an important component of ERA. Oxidative radicals are responsible for the dysregulation of many cellular functions and for damage to molecules, including DNA (reviewed by Cadet et al., 2010). As a consequence, the recent developments in Comet assay protocols combining enzymes involved in the repair of oxidative DNA damage are breaking ground to link toxicant-induced oxidative stress and DNA damage (see Collins, 2009, 2014, and references therein).

Studies attempting to integrate DNA damage retrieved from the *in vitro* Comet assay and biomarker responses of field-collected animals are lacking, which constitutes a gap within the validation of cell-based assays in ERA, despite the acknowledged importance of genotoxicity as a line-of-evidence (LOE). The present study aims essentially at comparing the performance, as ecotoxicological indicators, of the formamidopyrimidine-DNA glycosylase (FPG)-modified Comet assay in HpG2 cells exposed to sediment-bound contaminants with that of common oxidative stress-related biomarkers determined in three distinct organisms collected from an impacted estuarine area. Ultimately, it was intended to contribute for the validation of the data produced by the *in vitro* Comet assay as a LOE in ERA strategies. For this purpose, the present study integrates and re-interprets the findings from recent research on the Sado Estuary (SW Portugal), taken as the case study, and presents for the first time data from the *in vitro* analysis of sediment extract fractioning.

## MATERIALS AND METHODS

### STUDY AREA AND SAMPLE COLLECTION

**FIGURE 1:**
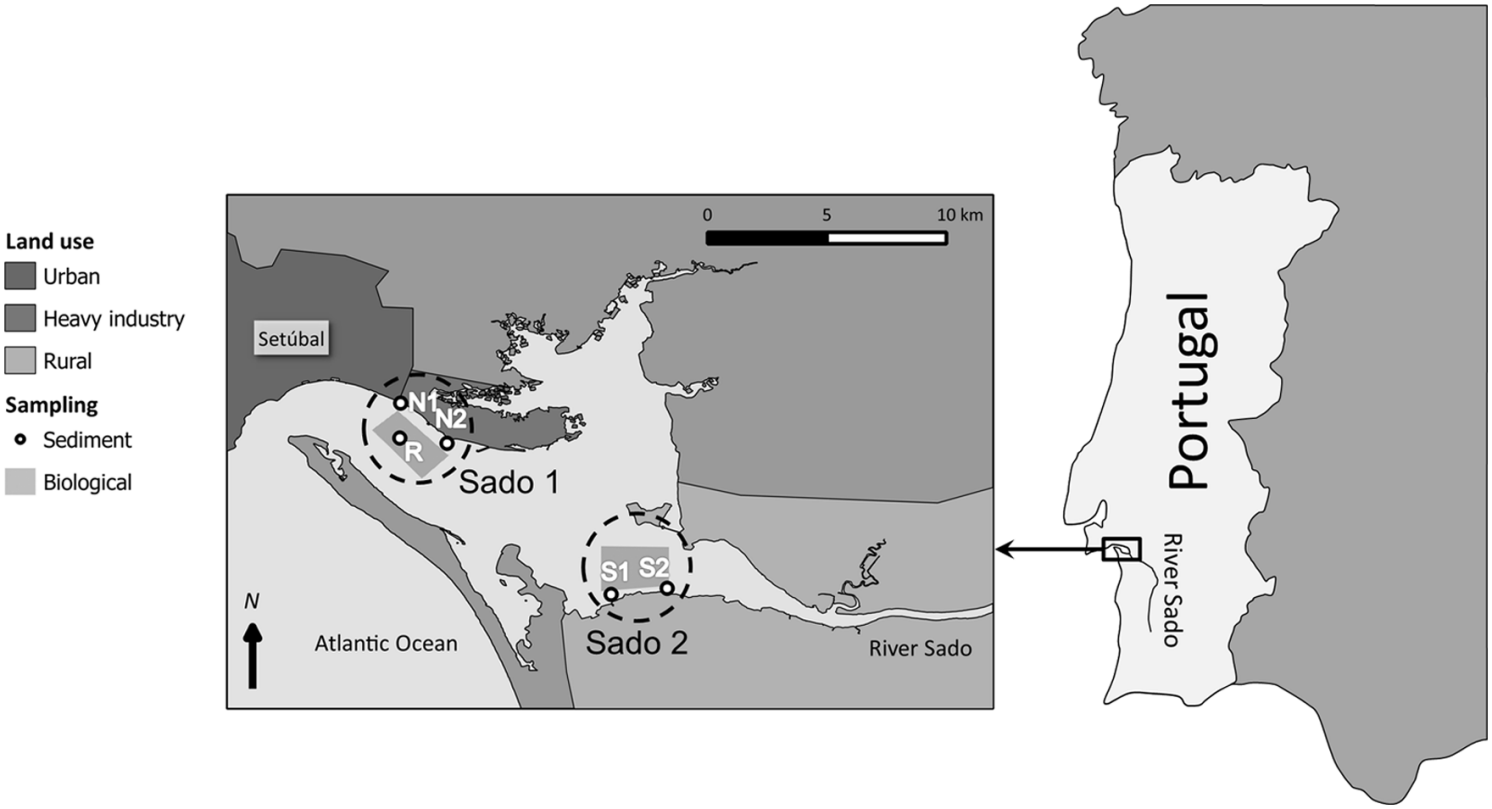
**Map of the river Sado Estuary, Portugal, highlighting the two study areas: Sado 1 (north) and Sado 2 (south).** Sediment collection sites and organism fishing grounds for each area are also indicated. Refer to the legend for specifics.

The Sado estuary, located in SW Portugal, consists of a large basin of high ecological and socio-economical importance. The estuary is very heterogeneous, with respect to its biogeography and anthropogenic use. The basin includes the city of Setúbal, with its harbor and heavy-industry belt, located in the northern area (Sado 1). On its turn, the southern region (Sado 2), where the mouth of the river Sado is situated, is essentially agricultural (**Figure 1**). Part of the estuary is classified as a natural reserve and, besides industry and shipping, the estuary is also very important for tourism, fisheries, and aquaculture. The river itself transports to the estuary fertilizers, pesticides from run-offs of the agriculture grounds upstream and metals from pyrite mining areas. The estuary has been judged to be globally moderately impacted by pollutants albeit ecotoxicologically diversified (refer to Caeiro et al., 2009, Costa et al., 2012, and references therein). Altogether, the multiple human activities result in diverse sources of contamination (most of which diffuse) and dictate the need to develop effective environmental managing and land use plans that include monitoring the presence, fate and effects of potential pollutants.

Sediment samples were collected from five different sites within the Sado estuary between spring 2007 and spring 2010. Sites N1 and N2 (Sado 1) are located off Setúbal’s harbor and industrial belt, respectively. Sites S1 and S2 (Sado 2), in the southern part of the estuary are located near an agricultural region with direct influence from the River Sado (**Figure 1**). The reference sediment (R) was collected from a sandy shellfish bed with high oceanic influence, from where clams were collected (see Carreira et al., 2013). Metallic/metalloid and organic toxicants (PAHs and organochlorines) were analyzed in sediments by means of inductively-coupled plasma mass spectrometry (ICP-MS) and gas chromatography–mass spectrometry techniques, respectively, with the results being validated through the analyses of certified reference materials (refer to Costa et al., 2011 and Carreira et al., 2013, for procedural details). Clams (*Ruditapes decussatus*) were collected from sites R and S1 upon sediment collection. Fish (*Solea senegalensis*), and cuttlefish (*Sepia officinalis*) were collected from acknowledged fishing grounds in Sado 1 and Sado 2 (**Figure 1**). Fish and cuttlefish biomarkers were contrasted to data of animals collected outside the estuary, within the same geographical region. However, sediment analyses (for pollutants, grain size, redox potential, and organic matter) from this external area yielded similar results to that of sediment R, which was found to be essentially devoid of any significant contamination, in spite of its proximity to sites N1 and N2. For such reason, oxidative stress biomarker data from fish and cuttlefish were geographically allocated to site R, for computational purposes. In order to congregate sediment toxicant levels into more manageable indices, these data were used to estimate sediment quality guideline quotients (SQG-Qs) for each class of contaminants and for total contamination, according to Long and MacDonald (1998), following contrasting to the probable effects level (PEL) guidelines for marine pollutants, available for most analyzed substances (MacDonald et al., 1996). The SQG-Q scores provide a measure of risk, allowing sediments to be classified as unimpacted if SQG-Q < 0.1; moderately impacted if 0.1 < SQG-Q < 1 and highly impacted if SQG-Q > 1 (MacDonald et al., 2004). **Table 1** summarizes the main sediment contamination data and respective SQG-Qs. Sediment data were retrieved from Costa et al. (2011) and Carreira et al. (2013).

**Table 1:** Sediment contamination data and respective sediment quality guideline quotients (SQG-Qs) per sediment sample.

		Area	Sado 1			Sado 2			
		Site	R*	N_1_**	N_2_*	S_1_*	S_2_*
Metal (μg/g)					
Metalloid		As	0.34 ± 0.26	23.98 ± 0.48	19.7 ± 5.21	26.44 ± 2.68	25.02 ± 8.84
		Se	1.84 ± 0.84	1.21 ± 0.02	1.92 ± 1.45	0.59 ± 0.21	0.72 ± 0.08
Metal		Cr	2.36 ± 0.36	80.73 ± 1.61	77.67 ± 4.57	62.22 ± 4.45	87.61 ± 2.97
		Ni	4.10 ± 1.66	33.30 ± 0.67	16.67 ± 1.1	17.15 ± 1.21	22.79 ± 9.47
		Cu	4.51 ± 1.05	172.72 ± 3.45	178.64 ± 7.01	74.15 ± 13.16	92.3 ± 5.63
		Zn	13.10 ± 1.51	364.83 ± 7.30	327.51 ± 1.16	269.79 ± 7.81	385.11 ± 35.69
		Cd	0.03 ± 0.02	0.26 ± 0.01	0.27 ± 0.03	0.33 ± 0.13	0.43 ± 0.19
		Pb	3.50 ± 0.48	55.19 ± 1.10	56.45 ± 3.1	25.3 ± 0.91	32.7 ± 1.21
Organic (ng/g)
		tPAH	19.60 ± 3.33	1 365.20 ± 232.08	1.076.98 ± 183.09	215.03 ± 36.55	82.47 ± 14.02
		tDDT	0.02 ± 0.00	0.37 ± 0.06	1.22 ± 0.21	0.21 ± 0.04	0.13 ± 0.02
		tPCB	0.05 ± 0.01	7.91 ± 1.34	5.37 ± 0.91	0.26 ± 0.04	0.27 ± 0.05

SQG-Q		SQG-Q_metal_	0.04	0.79	0.68	0.62	0.49
		SQG-Q_organic_	0.00	0.09	0.06	0.01	0.00
		SQG-Q_total_	0.02	0.33	0.37	0.31	0.25
Impact status			Unimpacted	Moderate	Moderate	Moderate	Moderate

*data from Carreira et al. (2013); **data from Costa et al. (2011).

### SEDIMENT EXTRACTS

Sediment contaminant extraction follows the protocol of Šrut et al. (2011), with few modifications, as described in detail by Pinto et al. (2014b). In summary, pulverized dry sediment samples were subjected to mechanical extraction with a series of organic solvents of increasing polarity. Fraction 1 (the crude extract) was obtained with a dichloromethane (DCM):methanol (2:1) mixture to attempt extraction of the bulk toxicants; fraction 2 with *n*-hexane (apolar); fraction 3 with DCM, and fraction 4 with methanol (the most polar solvent). The solvents were afterward evaporated at 45°C and the extracts reconstituted in dimethyl sulfoxide (DMSO). The concentrations of the extracts were estimated as mg sediment equivalent (SEQ) per mL of cell culture medium.

### *IN VITRO* ASSAYS

The human hepatocellular carcinoma cell line (HepG2) was obtained from the American Type Culture Collection (ATCC ref. HB-8065) and cultured as described in Pinto et al. (2014a,b). Cytotoxicity was measured through the neutral red (NR) assay, performed in triplicate for each experimental condition, as previously described (Pinto et al., 2014b). Briefly, after a 48 h exposure period to sediment extracts (from 5 up to 200 mg SEQ/mL), HepG2 cells were incubated with NR (3 h), which was afterward recovered and measured spectrophotometrically (540 nm). The relative cell viability, expressed as the percentage of viable cells, was estimated by the ratio between the mean absorbance of treated and control cells, assuming the mean absorbance of the negative control to represent 100% viable cells. The level of DNA damage and oxidative DNA damage was evaluated by the Comet assay and FPG-modified Comet assay, respectively, the latter to convert oxidized purines into single-strand breaks (Collins, 2009). The experiment was performed in triplicate. In brief: following a 48 h exposure period to each sediments extract, HepG2 cells were washed, detached, embedded in low-melting point agarose (1% m/v) and spread onto duplicate gels per replicate. Cells were then lysed (for at least 1 h) before nucleoid treatment with FPG or buffer only (30 min, 37°C). DNA was allowed to unwind (40 min) before electrophoresis (0.7 V/cm, 30 min). After staining with ethidium bromide, one hundred randomly selected nucleoids were analyzed per experimental condition. The mean percentage of DNA in tail was taken as the final endpoint for being regarded as one of the most consistent Comet metrics (Duez et al., 2003).

### BIOMARKER APPROACH

The multiple oxidative stress-related biomarker responses in wild organisms were retrieved from Carreira et al. (2013), Gonçalves et al. (2013), and Rodrigo et al. (2013), for clam, sole, and cuttlefish, respectively. The molluscan digestive gland and fish liver were chosen as target organs for being analog organs and due to their role in the storage and detoxification of xenobiotics. The oxidative stress-related biomarkers investigated in the present study were lipid peroxidation and catalase (CAT) activity in clams; lipid peroxidation, catalase activity, and glutathione *S*-transferase (GST) activity in fish; lipid peroxidation, GST activity, total glutathione (GSHt), and reduced/oxidized glutathione ratio (GSH/GSSG) in cuttlefish. Details of the procedures can be found in Carreira et al. (2013), Gonçalves et al. (2013), and Rodrigo et al. (2013). Briefly: GSHt was determined as through the enzymatic recycling method, using a commercial kit (Sigma–Aldrich), following manufacturer instructions. The GSH/GSSG ratio was estimated following derivatization of subsamples with 2-vinylpyridine (Sigma–Aldrich), in order to obtain the GSSG concentration. The ratio was determined as GSH/(GSSG/2). The activity of GST was determined spectrophotometrically using commercial kit (Sigma–Aldrich), following the instructions from the manufacturer, by measuring the increase in absorbance at 340 nm during 5 min, using chloro-2,4-dinitrobenzene (CDNB) as substrate. Lipid peroxides were determined through the thiobarbituric acid-reactive species (TBARS) assay developed by Uchiyama and Mihara (1978) and adapted by Costa et al. (2011). Samples were homogenized in cold phosphate-buffered saline, PBS (pH 7.4, with 0.7% NaCl) and the supernatant was deproteinated with trichloroacetic acid, after which thiobarbituric acid was added and the samples incubated for 10 min in boiling water. The absorbance of reddish pigment was measured at 530 nm and quantified through a calibration curve using malondialdehyde bis(dimethylacetal), from Merck, as standard. CAT activity was measured spectrophotometrically (at 240 nm during 6–8 min at 30 s intervals) according to method of Clairborne (1985), being estimated as units (U) per mg protein. All biomarker responses were normalized to sample total protein, determined through the method of Bradford (1976). The biomarker data are summarized in **Table 2**.

**Table 2:** Mean biomarker data (±SD) analyzed in the present work, for each species collected from the three study areas: Sado 1 (north); Sado 2 (south), and Reference.

		CAT (U/mg protein)	GST (nmol/min/mg protein)	GSHt (nmol/mg protein)	GSH/GSSG	LPO (nmol/mg protein)
**Area**						
Sado 1						
	Clam	24.75 ± 22.37				0.002 ± 0.001
	Fish	24.54 ± 21.94	0.12 ± 0.11			1.76 ± 1.05
	Cuttlefish		0.005 ± 0.002	0.11 ± 0.13	2.08 ± 2.39	0.69 ± 0.38
Sado 2						
	Clam	33.37 ± 27.84				0.003 ± 0.002
	Fish	46.91 ± 26.23	0.31 ± 0.14			1.26 ± 0.72
	Cuttlefish		0.003 ± 0.001	0.04 ± 0.08	2.22 ± 2.02	0.57 ± 0.32
Reference					
	Clam	18.70 ± 9.39				0.001 ± 0.000
	Fish	25.34 ± 20.64	0.21 ± 0.09			1.05 ± 0.52
	Cuttlefish		0.002 ± 0.001	0.04 ± 0.03	2.82 ± 1.73	0.23 ± 0.09

*Data from clam (*Ruditapes decussatus*), sole (*Solea senegalensis*), and cuttlefish (*Sepia officinalis*) were retrieved from Carreira et al. (2013), Gonçalves et al. (2013), and Rodrigo et al. (2013), respectively.*

### EC_50_ ESTIMATION

The half-maximal effective concentration (EC_50_) for cytotoxicity and genotoxicity was estimated for crude and fractionated extracts to allow the comparison of their relative cytotoxic and genotoxic potencies (see Seitz et al., 2008). Genotoxicity EC_50_ (with and without FPG treatment) was estimated by considering the highest measured %DNA in tail throughout the experiments as the maximal effect, since the %DNA in tail should not reach 100%. The EC_50_ values were estimated from normalized data through log-logistic regression and were computed using Stat4Tox 1.0 (Joint Research Centre of the European Commission), built for the R platform (Ihaka and Gentleman, 1996), version 2.10. Estimates are provided as mg SEQ/mL ± 95% confidence intervals.

### INTEGRATED BIOMARKER RESPONSE

The integrated biomarker response (IBR) indice was computed to integrate oxidative-stress biomarker responses determined in cuttlefish digestive gland (GST, GSHt GSH/GSSG, LPO), flatfish liver (CAT, GST, LPO), and clam digestive gland (CAT, LPO), according to the method described by Beliaeff and Burgeot (2002). Accordingly, the IBR is based on the partial score (*S*) estimates for each biomarker and organism. The scores were used to calculate the area (*A*) connecting consecutive coordinates (data points) in star plots. The IBR for each area (Sado 1, Sado 2, and the reference scenario) and *S* for each species were then calculated through the sum of the respective *A* values. See Rodrigo et al. (2013) for further details. The modifications suggested for IBR calculations, specifically the transformation to IBR/number of biomarkers (e.g., Broeg and Lehtonen, 2006), were not applied since for every area the same organisms and biomarkers were analyzed.

### STATISTICS AND INTEGRATION OF DATA

Data were mapped through a geographical information system (GIS) approach using QGis 2.0 and the digital map for mainland coastal waters (EPSG:4326 – WGS 84 coordinate system) made available by the Hydrographic Institute of the Portuguese Navy (http://www.hidrografico.pt). In order to obtain a general overview of the sediments’ contamination status, SQG-Q values for total contamination, metals, and organic toxicants were used for the analysis. The approach included also the EC_50_ estimates obtained from the Comet assay data (with and without FPG treatment) plus the global IBR for each area (combining all species and biomarkers). Interpolation of data points to raster layers was achieved through the inverse distance weight (IDW) algorithm from minimum–maximum normalized values.

Cluster analysis was done using Cluster 3.0, integrating SQG-Qs, EC_50_ estimates from the Comet assay and IBR values. Dendrograms and heatmaps were plotted using Java TreeView 1.1.6. Additional correlation statistics (Spearman’s *R*) and the Kruskall–Wallis Median Test adaptation for multiple comparisons (following recommendations by Duez et al., 2003) were computed with Statistica 8.0 (Statsoft).

## RESULTS

The cytotoxicity of the different extracts, as evaluated by EC_50_ estimates (**Table 3**), was highly variable. All extracts from the reference sediment (R) failed to yield significant cytotoxicity at the tested concentrations. Similar results were obtained for fractions 2 and 3 of any sediment. The lowest EC_50_ estimates, indicating higher cytotoxic potency, were obtained for fraction 1 (crude extract) of samples N1 and N2 (Sado 1 area). The cytotoxicity data were used to select the dose-range for genotoxicity testing, in order to avoid interference from cytotoxic events causing DNA strand breakage.

**Table 3:** Cytotoxicity EC_50_ estimates for HepG2 cells exposed to each extract fraction for all surveyed sediment samples (in mg SEQ/mL).

	Extract fraction			
Site	1	2	3	4
R	–	–	–	–
N1	39.8 (34.3–45.2)	n.a.	n.a.	n.a.
N2	88.7 (82.1–95.4)	–	–	265.3 (158.5–372.0)
S1	180.0 (162.7–197.3)	–	–	–
S2	223.5 (152.5–294.6)	–	–	160.9 (70.1–251.8)

*[–], not computable (effect too low); n.a., data not available; fraction 1, dichloromethane:methanol (crude extract); fraction 2, *n*-hexane; fraction 3, dichloromethane; fraction 4, methanol; ranges indicate the lower and upper 95% confidence limits.*

**FIGURE 2:**
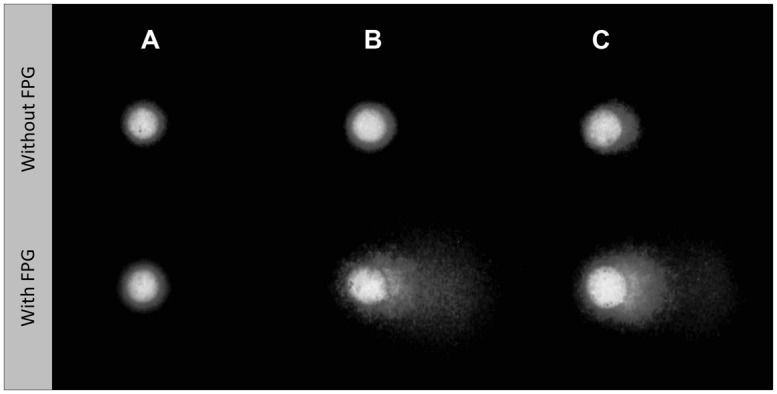
**Representative HepG2 Comet nucleoids treated without or with formamidopyrimidine-DNA glycosylase (FPG), to reveal oxidative damage to DNA. (A)** Negative control (dimethyl sulfoxide, DMSO only). **(B)** Cells exposed to the crude extract from sediment S1 [100 mg sediment equivalent (SEQ)/mL]. **(C)** Cells exposed to the crude extract from sediment S2 (200 mg SEQ/mL).

**FIGURE 3:**
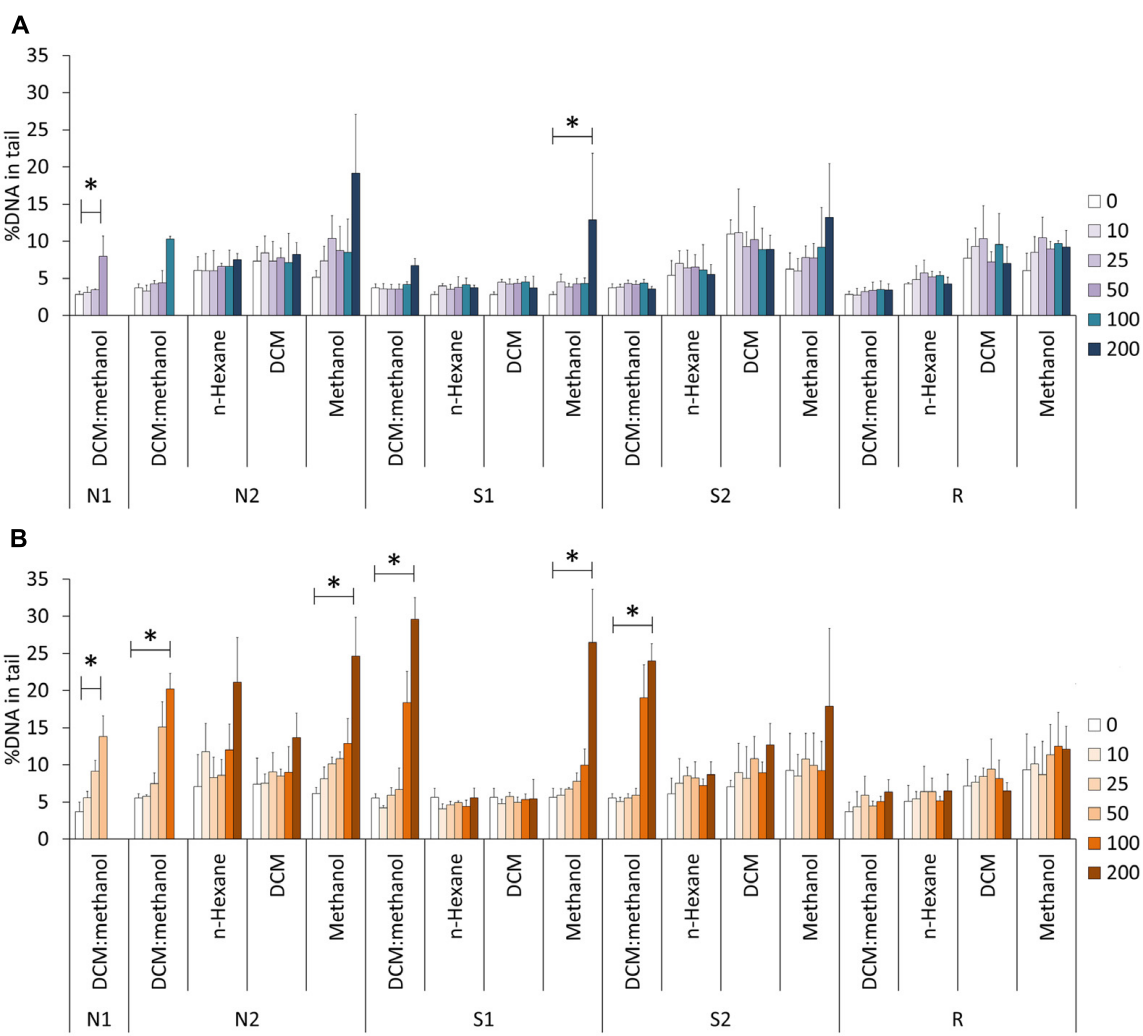
**Comet assay results in HepG2 cells exposed to the different sediment extracts at different concentrations (0–200 SEQ mg/mL). (A)** Experiments without FPG treatment. **(B)** Results from the FPG-linked Comet assay. *Indicates significant differences between multiple concentrations (Kruskall–Wallis Median Test, *p* < 0.05). The results are indicated as mean %DNA in tail ± SD. Dada from DCM:methanol extracts were retrieved from Pinto et al. (2014a). The concentration 0 mg SEQ/mL corresponds to the negative (solvent) control (DMSO only).

Examples of Comet nucleoids from exposed HepG2 cells are given in **Figure 2**. Non-oxidative strand breakage (**Figure 3A**) tended to increase with extract concentration, especially following exposure to extract fractions 1 and 4. Overall, DNA strand breakage was accentuated by the FPG-linked Comet assay (**Figure 3B**). The increase in total DNA damage in FPG-treated HepG2 cells was more pronounced following exposure to extracts S1 and S2 (especially fractions 1 and 4), attaining approximately 30% of DNA in the nucleoids’ tail. Conversely, no sizable effects were observed in cells exposed to any of the extracts from sediment R.

The EC_50_ estimates for DNA strand breakage revealed distinct trends between estuarine areas, sediment samples, and oxidative/non-oxidative damage (**Table 4**). Cells exposed to the crude extracts of Sado 1 sediment samples N1 and N2 yielded the lowest EC_50_ estimates for both FPG-treated and non-treated samples (meaning higher DNA damage at similar SEQ). In general, the FPG-modified Comet assay, which includes oxidative damage, resulted in decreased EC_50_ estimates. Furthermore, comparing data from the FPG-modified Comet assay to the conventional assay evidenced that the highest increase in oxidative DNA strand breakage occurred following exposure to sediment extract S1, fraction 1 (resulting in EC_50_ reduction by almost fourfold), and S2, fraction 1 (DCM:methanol) as well, for which no computable EC_50_ could even be retrieved from the conventional Comet assay. Overall, fractions 2 (*n*-hexane) and 3 (DCM) failed to produce estimates due to low induction of genotoxic effects. No EC_50_ values could be estimated from data of cells exposed to any of the fractions from the reference sediment (R). No correlations were found between cytotoxicity EC_50_ and DNA strand breakage EC_50_ estimates, with or without FPG-treatment (Spearman’s R, *p* > 0.05).

**Table 4:** DNA damage EC_50_ estimates (retrieved from the % of DNA in tail) for HepG2 cells exposed to each extract fraction for all surveyed sediment samples (in mg SEQ/mL) relatively to the maximum observed %DNA in tail throughout the study (≈30%).

	Extract fraction			
Site	1	2	3	4
*Alkaline Comet*				
R	–	–	–	–
N1	82.0 (34.8–129.1)	n.a.	n.a.	n.a.
N2	131.6 (103.6–159.6)	–	–	195.6 (19.1–374.1)
S1	364.5 (238.2–490.7)	–	–	223.9 (168.4–279.4)
S2	–	–	–	–
*Alkaline Comet*+* FPG*				
R	–	–	–	–
N1	65.4 (59.6–71.2)	n.a.	n.a.	n.a.
N2	72.6 (53.2–91.9)	175.5 (99.0 – 252.0)	354.6 (86.5–622.7)	127.8 (72.3–183.3)
S1	97.1 (90.2–104.0)	–	–	136.4 (117.4–155.4)
S2	104.1 (73.0–135.2)	–	–	–

*[–], not computable (effect too low); n.a., data not available; fraction 1, dichloromethane:methanol (crude extract); fraction 2, *n*-hexane; fraction 3, dichloromethane; fraction 4, methanol; ranges indicate the lower and upper 95% confidence limits.*

**FIGURE 4:**
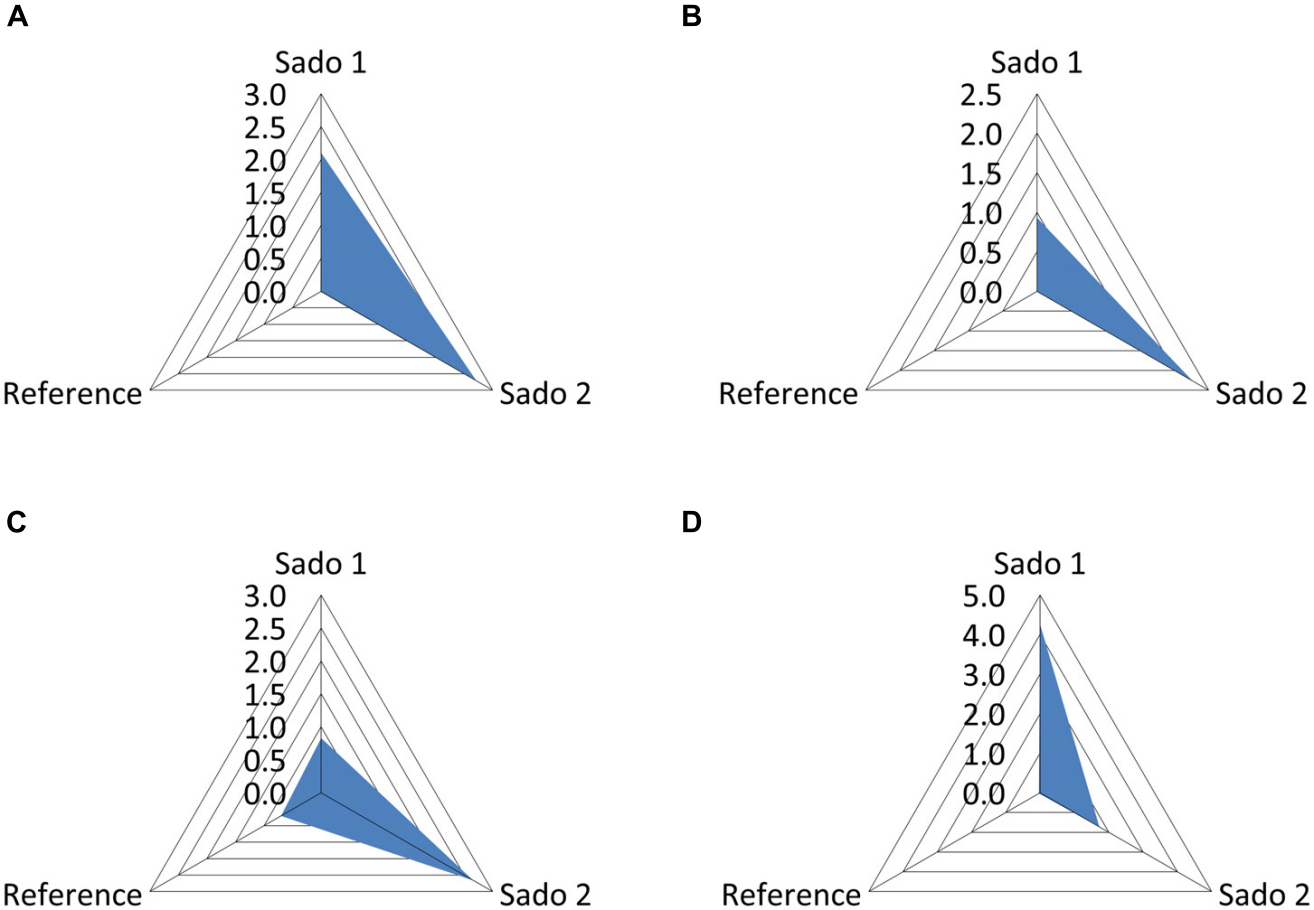
**Plots of the integrated biomarker response (IBR) for the three areas, Sado 1 (urban and industrial), Sado 2 (riverine and agricultural) and Reference. (A)** Global IBR combining clam, fish (sole), and cuttlefish; IBR scores (*S*) for clam **(B)**; fish **(C)**, and cuttlefish **(D)**.

Clam, fish, and cuttlefish yielded distinct patterns of oxidative biochemical damage (measured through lipid peroxidation) and responses to oxidative stress (see **Table 2**). In accordance, distinct IBR scores were obtained from each surveyed species. However, the aggregated results indicate a similar trend to increase oxidative stress responses and effects in animals collected from the impacted sites Sado 1 (IBR = 2.10) and Sado 2 (IBR = 2.72), compared to the reference scenario (IBR = 0.01), when combining all three species (**Figure 4A**). Clams, for which lipid peroxidation and CAT activity were surveyed, yielded higher IBR scores for Sado 2 (**Figure 4B**), similarly to fish (**Figure 4C**), for which GST was added. Conversely, cuttlefish, for which lipid peroxidation, GST activity, GSHt, and reduced/oxidized glutathione ratio were surveyed, yielded higher IBR for Sado 1 (**Figure 4D**).

**FIGURE 5:**
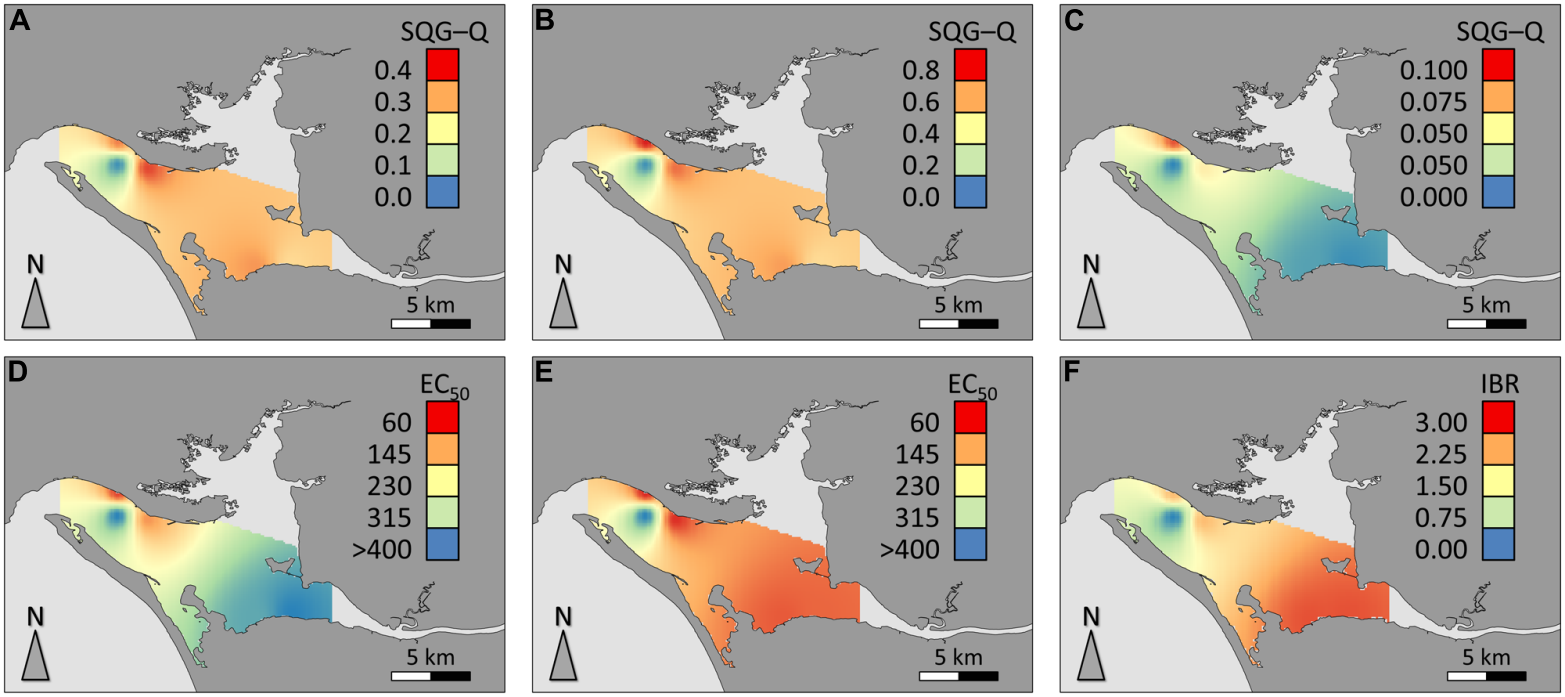
**Spatial distribution of data for the study area. (A)** SQG-Q for total sediment contaminants (metals plus organic); **(B)** SQG-Q for sediment metals; **(C)** SQG-Q for organic sediment contaminants; **(D)** HepG2 EC_50_ for DNA strand breakage (crude extract exposure); **(E)** HepG2 EC_50_ for oxidative DNA strand breakage (crude extract exposure); **(F)** IBR for oxidative stress-related biomarkers, all species combined (clam, fish, and cuttlefish). SQG-Qs and IBR are dimensionless. EC_50_ estimates are expressed as mg SEQ/mL.

**FIGURE 6:**
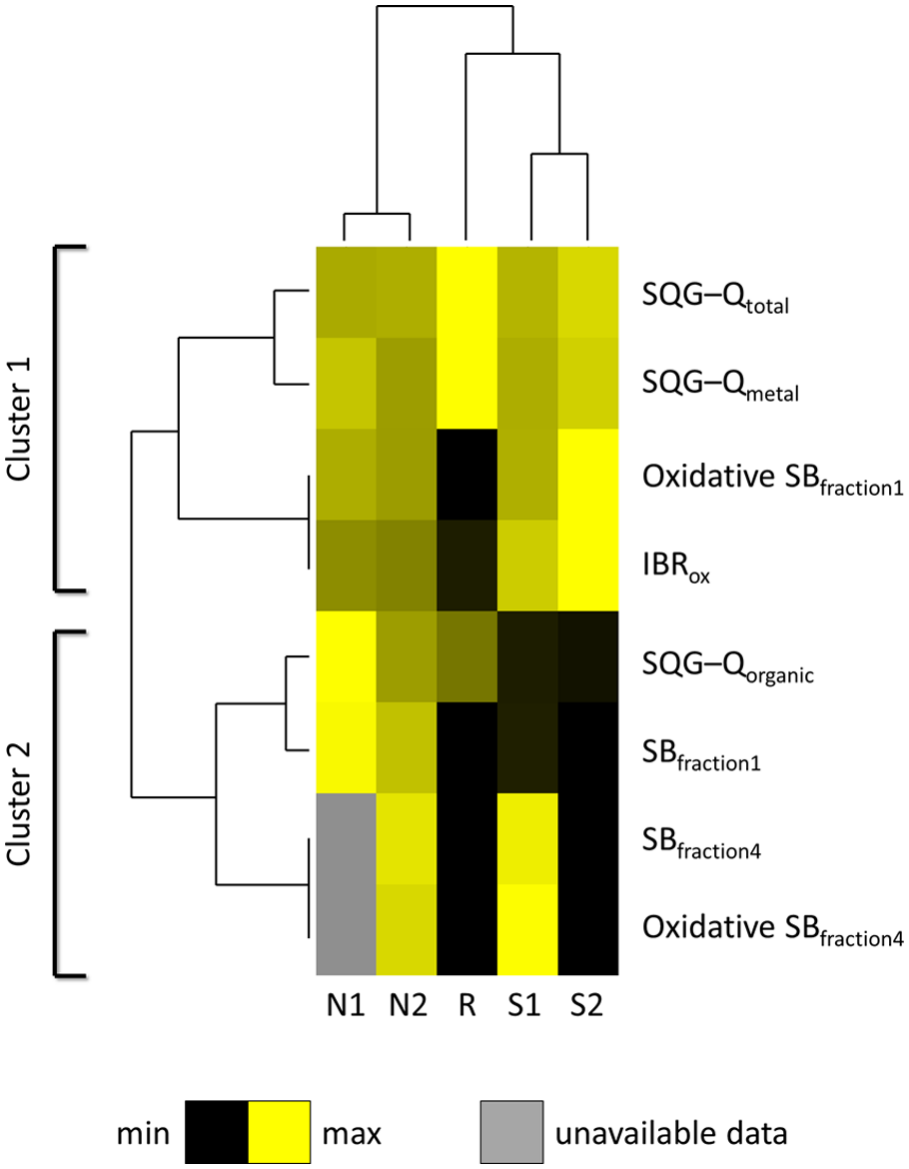
**Cluster analysis heatmap.** Analysis combines sediment collection sites (N1, N2, S1, S2, and R) plus SQG-Q scores for sediment contaminants (total, metal, and organic pollutants) and biological responses: DNA strand breakage (SB), oxidative and non-oxidative, inputted as 1-EC_50_ relatively to the highest %DNA in tails from the study), for HepG2 cells exposed to fractions 1 (crude extract, DCM:methanol extraction) and 4 (methanol extraction only), plus IBR for oxidative stress biomarkers combining clam, fish, and cuttlefish (IBR_ox_). Clustering between endpoints was achieved taking Spearman’s rank-order correlation *R* as distance metric. Clustering between sites was obtained with Euclidean distances. Complete linkage as employed as amalgamation rule for the dendrograms.

Spatial distribution of data for sediment contamination plus Comet assay and IBR results are presented in **Figure 5**. The distribution of sediments contaminants was found to be very heterogeneous within the estuary, marking a distinction between Sado 1 (urban and industrial) and Sado 2 (rural and riverine) areas (**Figures 4A–C**), with the reference site evidencing a clear distinction from its immediate surroundings. The distinction between Sado 1 and Sado 2 is more obvious for organic contaminants, of which PAHs (**Figure 5C**) are the most representative (see **Table 1** also). These contaminants were best represented in Sado 1 sediments N1 and N2, in line with the findings retrieved from the conventional Comet assay (**Figure 5D**). Oxidative DNA strand breakage increased most notoriously in HepG2 cells exposed to sediments from Sado 2 (**Figure 5E**). Accordingly, animals from Sado 2 yielded comparatively the highest combined IBR value for oxidative stress-related biomarkers (**Figure 5F**). In agreement with the spatial distribution of data, cluster analyses combining sediment and biological data grouped sites N1 and N2 within the same cluster, both belonging to Sado 1 whereas sites S1 and S2 (Sado 2) constituted a clearly distinct group. Still, the Reference site (R) exhibited a closer relation to Sado 2 than to Sado 1 sites (**Figure 6**). Oxidative DNA damage caused by exposure to fraction 1 was best correlated to IBR and, together with SQG-Qs for metals and total toxicants, formed a distinct cluster from the one (cluster 2) comprising SQG-Qs for organic toxicants, non-oxidative DNA damage, and oxidative DNA damage resulting from exposure to the extract fractions 4 (methanol).

## DISCUSSION

The present work showed that estuaries, even if regarded as moderately impacted, may be highly heterogeneous with respect to the distribution of pollutants which, consequently, is translated into a complex pattern of biological effects and responses to toxicants. Oxidative DNA damage was found to be associated to IBR estimates (**Figure 6**), for oxidative stress biomarkers analyzed in local species (combining clams, fish, and cuttlefish). This indicates a relationship, as potential lines-of-evidence, between two distinct sets of oxidative effects, i.e., biochemical and genetic, determined in wild animals, and HepG2 cells, respectively.

Overall, the results indicate that oxidative effects endured by wild organisms and HepG2 cells are better associated either to total contamination or to metals (the best represented toxicants), than to well-known genotoxicants like PAHs. It must be emphasized that sediment contamination, with particular respect to organic contaminants (among which PAHs are the best represented), was globally higher in sediments N1 and N2 (i.e., from the industrial area of the estuary). Nonetheless, the increment of DNA strand breakage in FPG-treated cells relatively to the standard assay was higher in HepG2 cells after exposure to extracts from Sado 2 (the rural and riverine area), indicating a higher level of oxidative DNA damage. This observation is accordant with the present IBR results and also as disclosed by the original research with cells with unfractionated extracts (see Pinto et al., 2014a for further details). In fact, under this scope, the analyses with this cell line provided a globally more conclusive distinction between contaminated and reference areas than each species individually, since clams, fish, and cuttlefish yielded different results (**Figure 4**). However, any potential link between oxidative DNA damage in cells and biochemical oxidative stress in wild organisms remains elusive, since organisms hold specific abilities to cope with exposure to toxicants and the oxidative stress hitherto derived.

Past research to determine the effects of sediment contamination in the Sado Estuary based on a multi-biomarker approach in soles exposed *in situ* and *ex situ* revealed that the *in vivo* Comet assay provided one of the most consistent measurements to distinguish contaminated from non-contaminated sites, among a wide battery of biomarkers (Costa et al., 2012). Oppositely, Gonçalves et al. (2013) disclosed that anti-oxidative defenses, namely the activity of CAT and GST were inhibited in fish from Sado 1, where highest lipid peroxidation levels occurred. These findings are thus accordant with reduced IBR scores in animals from Sado 1 (**Figure 4C**). The same authors then hypothesized that one of the factors involved in such inhibition was the complex interaction of toxicants (organic and metallic). Altogether, when comparing the effects on fish and human cells, it may be inferred that oxidative stress occurs indeed as a consequence of exposure to toxicants from Sado 1, whether translated into oxidative DNA lesions or biochemical damage. This information is in agreement with higher levels of contamination by organic compounds, especially PAHs, since metals presented similar values between the two main areas of the estuary (**Table 1**). On the other hand, molluscs provided consistent, albeit opposite, responses that are related to habitat and behavior. Clams (sedentary burrowers) from Sado 1 were collected from the precise same site than sediment R (the “clean” reference sediment); so, not surprisingly the IBR score was lower in comparison to clams collected from Sado 2 (specifically, from site S1). On its turn, cuttlefish (a foraging, territorial, predator) was consistently responsive to background contamination of Sado 1. Yet, these animals are a novelty within the field of research and little is known about its physiological responses to chemical challenge (see Rodrigo et al., 2013, for details).

The current findings are partially accordant with those obtained by Šrut et al. (2011) and Pinto et al. (2014b), who revealed higher strand breakage in a fish and human hepatoma cell line, respectively, exposed to crude extracts (dichloromethane:methanol) of marine sediments, when compared to exposure to fractions obtained with increasingly polar solvents. In fact, the significant correlations between EC_50_ estimates (oxidative and non-oxidative DNA damage) and SQG-Qs for organic and inorganic toxicants indicate that this extraction method was efficient for the bulk of toxicants (**Figure 6**). However, in the present study, fractions 2 (*n*-hexane) and 3 (dichloromethane) yielded only marginal results. Considering that metals are indeed the most significant toxicants determined in Sado sediments from contaminated areas, the results are in line with SQG-Qs (**Table 1**), since exposure to fraction 2 should mean exposure to PAHs and other highly hydrophobic substances. Moreover, it was observed that sediments from Sado 2 (S1 and S2) account primarily for oxidative DNA damage in HepG2 cells, showing that distinct sets of sediment toxicants were retrieved from both Sado areas (**Figure 2**; **Table 3**). Most likely, Sado 2 sediments contain important levels of more hydrophilic toxicants, such as metals and potentially unsurveyed organic substances, either able to cause oxidative DNA damage or some type of alkylating lesions that might have been converted in strand breaks following FPG treatment (see Collins, 2014).

It must be noted that HepG2 cells have already been found sensitive to metal-induced DNA strand breakage measurable by the standard Comet assay, albeit yielding non-linear cause-effect relationships likely due to adequate deployment of defenses such as metallothioneins (Fatur et al., 2002). These findings have been confirmed through the exposure of HepG2 cells to metals extracted from soils (in aqueous phase), revealing, nevertheless, reduced sensitivity (Vidic et al., 2009). Still, unlike the present study, oxidative DNA damage was not measured in these works. The current results are also accordant with those obtained by Kammann et al. (2004), who subjected a fish cell line (from *Cyprinus carpio*) to extracts (also transferred to DMSO) from marine sediments and observed that extracts obtained with more polar solvents were more genotoxic (as determined through the standard Comet assay) than those obtained with *n*-hexane. The same authors discussed that reduced metabolic activation could, at least in part, contribute to explain the results. As such, it is possible, though, that enhanced metabolic activation in HepG2 cells could have rendered more significant results for the tests with fractions 2 and 3 (prepared with more hydrophobic solvents) than actually measured (**Table 4**), even though these cells are generally acknowledged to retain the mechanisms involved in PAH bioactivation (with production of ROS as by-products) by CYP mixed-function oxidases (Knasmüller et al., 2004). However, inefficient extraction cannot be definitely excluded. The current results for fraction 1 (crude extract) are more indicative of metal-induced genotoxic effects (oxidative and non-oxidative), which is in good agreement with the results from the cluster analyses and the overall contamination pattern of sediments (**Figure 6**). It must also be noticed that cytotoxicity in HepG2 cells exposed to the different extracts was not clearly related to DNA damage, which is in accordance with other works dealing with *in vitro* exposures to whole marine sediment extracts (e.g., Yang et al., 2010). The results indicate that the complex mixture of toxicants within the tested sediments, specifically fractions 1 and 4, elicit differential genotoxic and cytotoxic effects. It must also be stressed that the cytotoxic effects of solvents may be disregarded since, in all cases, the solvents were evaporated and replaced with DMSO.

There are indications that the standard alkaline Comet assay may be less sensitive to detect PAH-induced DNA lesions when compared, for instance, to the determination of adduct formation, inclusively in HepG2 cells (Tarantini et al., 2009). This information may leads to the hypothesis that PAH-induced non-oxidative DNA damage might have been underestimated in HepG2 cells exposed to the crude extracts from sediments N1 and N2. Even so, the FPG-modified Comet assay has been found to greatly increase the assay’s sensitivity when surveying environmental toxicants (Kienzler et al., 2012), which is accordant with the present findings (**Figure 3**; **Table 4**), particularly in HepG2 cells exposed to the crude and methanolic extracts. From the results, it may be inferred that sediment extract fractioning combined with the enzyme-modified Comet assay is a potentially valuable toxicity identification evaluation (TIE) strategy to monitor environmental genotoxicants, in the sense that by removing causative agents, cause–effect relationships may be sought through a break-down approach (see Chapman and Hollert, 2006). Nonetheless, this sort of methodology needs yet much research with respect to establishing causation, i.e., to determine toxicants and respective effects of exposure *in vitro* and *in vivo*.

Even though fish and mammalian cell lines have been found equally sensitive to test cytotoxic and genotoxic effects of environmental contaminants (Castaño and Gómez-Lechón, 2005), there are many differences between *in vitro* and *in vivo* bioassays that call for caution when direct comparisons are made, particularly if animals collected from the wild are being surveyed. Anti-oxidative stress responses in organisms are acknowledged to be complex and dependent of numerous factors, internal and external, of which toxicant concentrations in the environment account for just a few. Although the subject is not well understood in aquatic invertebrates, inhibition of anti-oxidant responses has been described in fish exposed to certain toxicants (like metals) or their mixtures (e.g., Atli et al., 2006; Elia et al., 2007; Costa et al., 2010). This premise was also highlighted by Gonçalves et al. (2013), in face of elevated lipid peroxidation and higher level of histopathological alterations in the livers of sole collected from Sado 1. Moreover, previous studies have showed that sediments from this same area caused DNA strand breakage *in vivo* through a series of *in* and *ex situ* bioassays performed with *S. senegalensis*, which further supports the present findings (refer to Costa et al., 2008, 2011). It is also noteworthy that metals, the most representative contaminants in the estuary, may be indirectly genotoxic by impairing DNA repair and anti-oxidant enzymes (see Leonard et al., 2004), which likely affected HepG2 cells. Still, the integration of biomarker responses of the three species yielded differentiation between an impacted estuarine environment and the reference scenario, consistent with DNA damage measured through the Comet assay in HepG2 cells exposed to sediment extracts. Altogether, the present findings illustrate the purposefulness and adequacy of multiple lines-of-evidence in ERA, namely combining field sampling of multiple species, multiple biomarkers and *in vitro* assays to evaluate genotoxicity. As upheld by Chapman et al. (2013), the use of different lines-of-evidence, especially if appropriately incorporated into integrative weight-of-evidence assessments for management decision making, can reduce uncertainty and therefore assist determining causation.

## CONCLUDING REMARKS

In the present work, an integrative assessment of genotoxic effects triggered by sediment-bound contaminants with oxidative stress biomarkers in three different species collected from an impacted estuary was conducted, consisting of an innovative combination of cell and whole-organism responses. The *in vitro* Comet assay (to determine oxidative or non-oxidative DNA damage) is an expanding tool in ERA, with the potential to become a LOE within its own right if proper validation through realistic case studies is achieved. Not dismissing the clear need to endeavor future research, the present work showed that the enzyme-modified Comet assay applied to HepG2 cells in a practical ERA context can yield results that are overall consistent and complementary with oxidative stress biomarkers analyzed in field-collected organisms. As such, the deployment of the *in vitro* Comet assay in human carcinoma cell lines and its combination with more traditional LOEs may meet its purpose even in scenarios where establishing cause–effect relationships is likely hampered by challenging circumstances such as the presence of complex mixtures of toxicants.

## Conflict of Interest Statement

The authors declare that the research was conducted in the absence of any commercial or financial relationships that could be construed as a potential conflict of interest.
